# Editorial: Sex, money, and romantic relationships

**DOI:** 10.3389/fpsyg.2023.1225763

**Published:** 2023-06-13

**Authors:** E. Jeffrey Hill, Ashley B. LeBaron-Black, Chelom E. Leavitt, Xiaomin Li

**Affiliations:** ^1^School of Family Life, Brigham Young University, Provo, UT, United States; ^2^Department of Applied Social Sciences, The Hong Kong Polytechnic University, Kowloon, Hong Kong SAR, China

**Keywords:** sexual satisfaction, relationship satisfaction, personal finance, family finance, sex, money, romantic relationships, marriage

In this editorial, we introduce a collection of articles for *Frontiers in Psychology* entitled “*Sex, money, and romantic relationships*.” We believe that these articles are a vanguard of the burgeoning research interest in studying these three topics together, and how they concurrently influence important financial, sexual, and relational outcomes.

Talking about sex and money publicly is taboo in most cultures. Perhaps relatedly, two of the primary drivers of couple conflict and relational dissolution/divorce are also sex and money. Sex and money are hard to talk about but are central to most individuals and romantic relationships. They are also reflective of core attitudes and values. The academic domains of family finance and sexuality are both growing rapidly, and each is vital to our understanding of romantic relationships. While studying them together is not yet common, it has already yielded important findings. The aim of this Research Topic was to jump-start the process of conducting this type of cross-domain research.

We cast a wide net in soliciting manuscripts. The only pre-condition for submission was that the manuscript contain at least one variable in the area of finances, at least one variable in the area of sexuality, and at least one variable in the area of romantic relationships. Four manuscripts made it through the peer review process to publication, and these four covered diverse areas of our topic. We will briefly examine each one.

The first article (Saxey et al.) in our Research Topic is “*Latent profiles of sleep quality, financial management, and sexual satisfaction in emerging adult newlywed couples and longitudinal connections with marital satisfaction*.” Saxey et al. creatively named four latent profiles: flourishers (high-quality sleep, responsible financial behaviors, and high sexual satisfaction); financially challenged lovers (average sleep, less responsible financial behaviors, and high sexual satisfaction); drowsy budgeters (low-quality sleep, responsible financial behaviors, low sexual satisfaction); and flounderers (low-quality sleep, less responsible financial behaviors, low sexual satisfaction). The authors then used profile membership to predict marital satisfaction 1 year later, demonstrating that couples' intersection of these three variables jointly predicted marital wellbeing. An interesting global finding was that for newlyweds, high sexual satisfaction was more important than responsible financial behaviors in predicting relationship quality.

The second article (Dew et al.) in our Research Topic is “*Money lies and extramarital ties: Predicting separate and joint occurrences of financial deception and extramarital infidelity*.” For decades there has been a plethora of research documenting antecedents and outcomes of extramarital sexual affairs. In recent years, there have also been several articles examining marital financial infidelity (i.e., partners lying to each other about money). In this Research Topic, Dew et al. became the first to concurrently examine predictors of marital financial deception and extramarital sexual affairs. One interesting finding was that extramarital flirting was associated with not only having an extramarital sexual affair but also with engaging in marital financial deception. This study provides evidence that sexual and financial domains intersect within marriages.

The third article (Turner et al.) in our Research Topic is “*Economic distress and perceptions of sexual intimacy in remarriage*.” This study highlights that financial and sexual factors influence each other in remarriages and that the associations between financial factors and sexual factors may differ by gender. For example, Turner et al. found that wives' report of economic distress was directly related to their rejection of their partner's sexual advances, but this association was not found for husbands. It is also noteworthy that the sample was of remarried couples, which is an understudied sample in both the field of family finance and the field of sexuality.

The fourth and final article (Allsop et al.) in our Research Topic is “*Perceived financial burden is indirectly linked to sexual well-being via quality of life among couples seeking medically assisted reproduction*.” In this article, Allsop et al. focused on couples who were seeking medically assisted reproduction (MAR). They explored how income and the financial burden of seeking MAR were linked to sexual wellbeing. Infertility can cause considerable emotional and financial stress to individuals and, on a dyadic level, couple functioning. Results from this study demonstrate that both financial and sexual factors contribute to that stress and that these factors intersect in contributing to couple-level stress.

In this Research Topic “*Sex, money, and romantic relationships*,” we sought to increase collective understanding of how financial and sexual factors intersect in relationships. The four articles used a variety of samples: Saxey et al. used a dyadic, nationally representative sample of U.S. newlyweds; Dew et al. used a nationally representative sample of U.S. married adults; Turner et al. used a dyadic sample of U.S. newlywed remarried couples; and Allsop et al. used a dyadic sample of couples from the U.S. and Canada seeking MAR. Money and sex are two important areas of romantic relationships. This Research Topic provides evidence for how financial and sexual factors concurrently impact relational outcomes in a variety of relational circumstances. The four articles were also guided by a variety of theoretical frameworks: Saxey et al. and Allsop et al. used couples and finance theory (Archuleta and Burr, 2015), Dew et al. used social exchange theory (Thibault and Kelley, 1959), and Turner et al. used multidimensional family development theory (Crapo and Bradford, 2021). Thus, various theories can be used as lenses through which to consider the joint influence of financial and sexual variables in relationships. All four articles had a common thread: sexual and financial factors intersected in predicting relational outcomes. Together, these articles provide strong evidence that sexual and financial factors should be concurrently studied more frequently. We hope that this Research Topic of articles examining financial and sexual variables simultaneously will be just the beginning of a greater emphasis on this area.

## Author contributions

All authors listed have made a substantial, direct, and intellectual contribution to the work and approved it for publication.
